# Limited Effect of Management on Apple Pollination: A Case Study from an Oceanic Island

**DOI:** 10.3390/insects11060351

**Published:** 2020-06-04

**Authors:** Adara Pardo, David H. Lopes, Natalia Fierro, Paulo A. V. Borges

**Affiliations:** 1 cE3c-Centre for Ecology, Evolution and Environmental Changes/Azorean Biodiversity Group, Faculty of Agriculture and Environment, Department of Environmental Sciences and Engineering, Universidade dos Açores, PT-9700-042 Angra do Heroísmo, Açores, Portugal; david.jh.lopes@uac.pt (D.H.L.); 27nataliafifre@gmail.com (N.F.); paulo.av.borges@uac.pt (P.A.V.B.); 2 Forestry Research Group-INDEHESA, University of Extremadura, 10600 Plasencia, Spain

**Keywords:** conventional farming, diptera, herbaceous cover, hymenoptera, insect visitation, *Malus domestica*, organic farming, pollination services, species diversity

## Abstract

Intensive agricultural practices leading to habitat degradation represent a major threat to pollinators. Diverse management practices are expected to influence wild pollinator abundance and richness on farms, although their effect in perennial crops is still unclear. In this study, we assessed the impact of management on apple (*Malus domestica*) pollination on an oceanic island, by comparing conventional (with and without herbicide application) and organic apple orchards. Pollinator visitation and pan trap surveys were carried out in six apple orchards in Terceira Island (Azores) and the landscape composition surrounding orchards was characterized. We also quantified fruit set, seed set and apple weight. We found no significant effect of management on insect visitation rates, whereas there was a negative association with increasing surrounding agricultural land. In contrast, management had an effect on species abundance, richness and diversity at the orchard level. Conventional orchards without herbicides showed higher abundance than the rest, but lower richness and diversity than conventional orchards with herbicides. Management had an effect on fruit set, but not on seed set or fruit weight. Our results suggest that management alone is insufficient for the overall improvement of apple pollination on an oceanic island, while landscape composition may play a relevant role.

## 1. Introduction

Pollination is widely recognized as an essential ecosystem service and a vital process to sustain food security [1,2]. However, the impact of biodiversity erosion drivers, namely habitat loss and degradation, climate change, or the intensification of agriculture in the past half-century, has triggered a decline in wild and managed bees and other pollinating insects in several regions of the world [3,4,5,6,7,8]. Agriculture poses many threats to insect pollinators, including changes in land use, loss, fragmentation and degradation of habitat, introduction of exotic organisms, modern agricultural practices, and pesticide use [9,10]. The extensive use of insecticides (that can cause mortality by direct intoxication), herbicides and fertilizers (that can affect pollinators indirectly by decreasing floral resource availability) have been identified as the major drivers in the decline of pollinators [3] and other insects [11].

Diverse agricultural management practices are thus expected to influence wild bee abundance and richness on farms [12]. Organic farming, for instance, can improve bee abundance, richness and productivity [13,14,15], as long as sufficient habitat exists to maintain source populations [16,17]. However, the effect of management practices on insect pollination in perennial crops seems to be less consistent than in annual crops [18,19,20]. This could be a result of perennial systems usually having available a range of permanent structural elements such as hedgerows that can potentially attract pollinators, even under intensive management leading to low floral biodiversity [19].

Apple (*Malus domestica*) is one of the most important fruit crops globally, both economically and production-wise [21]. Apple, together with a majority of agricultural crops worldwide, is dependent on animal pollination [2]. The predominant pollination vector for apples are insects, such as bees (Insecta, Hymenoptera) and hoverflies (Diptera, Syrphidae). Their activity in orchards is thus essential for apple production globally [1,22,23]. In addition, insect pollinators have been shown to promote increased quality of apples [24] and pest management [25], which are indirect and more difficult benefits to measure, but extremely important for the agricultural market. Studies examining the effect of management practices on apple insect pollination are still scarce [19,20,26,27,28] and they report contrasting results. Available studies reported no differences in insect pollination between organic and conventional apple fields [19] or higher flower visitation rate and species richness in organic orchards in comparison to Integrated Pest Management (IPM) orchards [26]. In addition, results from studies assessing pollinator communities in conventional vs. abandoned or reduced-risk management apple orchards found either no differences [20,27] or higher richness and diversity in abandoned orchards [28]. Further studies examining these processes in apple cultivars are needed in order to draw patterns that are more general.

Additionally, there is still limited information on the condition of pollinators and the related ecosystem services they provide in oceanic islands, although recent research has demonstrated that insect pollinators play a key role in agricultural production [29]. Previous studies have shown that oceanic insular ecosystems usually support less complex pollination networks with lower numbers of pollinator species, and that these mostly comprised generalist species [30,31,32]. Thus, pollination networks on oceanic islands are probably the most vulnerable to disturbances [31], including threats from intensive agricultural practices. The profound changes islands have experienced over the last century are known to have impacted several components of island ecosystems [33] and diversity [34], but little is still known about the current status of island ecological networks, and in particular, of pollinating insects.

In this study, we assessed the impact of agricultural management on apple pollination on an oceanic island (Terceira, Azores, Portugal), by comparing conventional (with and without herbicide application) and organic apple orchards. Both organic farming practices which do not employ agrochemicals and intensive practices that maintain an herbaceous cover are expected to result in higher pollinator visitation rates, species diversity and pollination service provision than in intensive management orchards without herbaceous cover. Additionally, we evaluated the impact of the surrounding landscape composition on apple pollination. We expect that the prevalence of intensive agriculture may have deleterious impacts on pollinator diversity and activity.

## 2. Materials and Methods

### 2.1. Study Sites

**Figure 1:**
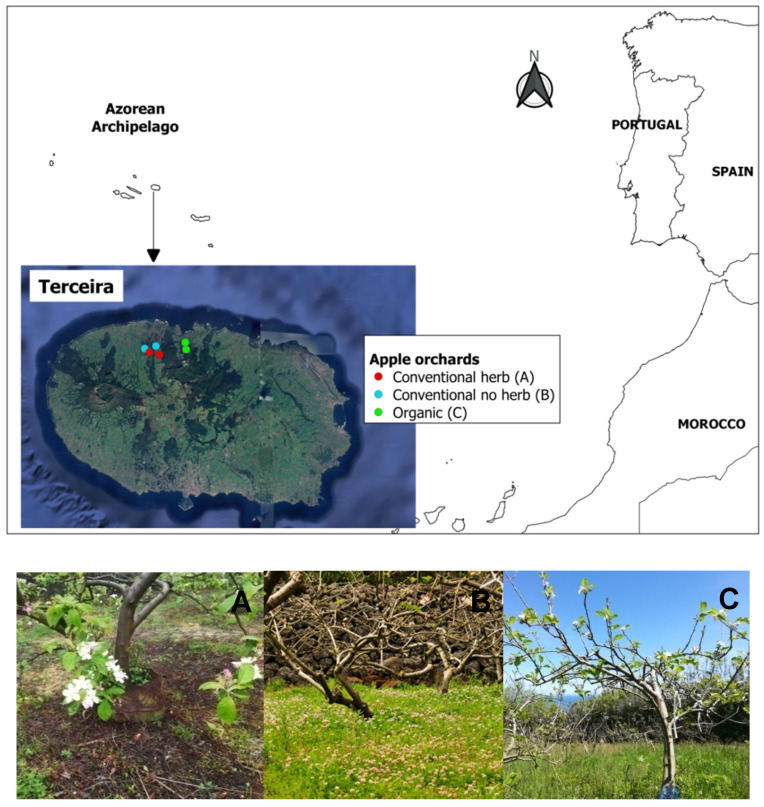
Location of the study sites in Terceira Island (Azorean archipelago) and example images of the three types of apple orchards studied, under different management systems: (**A**) Conventional with herbicide application; (**B**) Conventional without herbicide application; (**C**) Organic apple orchards.

This work was carried out in Terceira Island, within the Azores archipelago (Figure 1). The archipelago is located in the North Atlantic Ocean (38°37′ N–38°48′ N, 27°02′ W–27°23′ W) with an area 402 km^2^ and a maximum elevation of 1023 m. Apple orchards in Terceira account for a relatively small percentage of the territory and are interspersed between pastures, other crop fields and forests.

The study was conducted in six apple orchards (Figure 1) managed by different growers who implemented diverse management systems. There were no honeybee hives present in any of the studied orchards. Dominant apple varieties grown included Rennet, Royal Gala and Mutsu. Two orchards were managed in a conventional way, with the use of pesticides for pest and disease control, fertilizers and herbicides to remove herbaceous cover (“conventional with herbicides”). Two other orchards were managed in a conventional way making use of pesticides and fertilizers but without the use of herbicides, thus conserving herbaceous cover in the orchard (“conventional no herbicides”). The last two orchards were managed according to organic guidelines with no chemical inputs for pest control or fertilization (“organic” hereafter). Orchards were separated from each other by a minimum distance of 500 m. This minimum distance was considered greater than the average foraging range of most pollinator species found in the area [35,36].

The surrounding landscape composition was estimated within a 500m buffer around each orchard. The amount (ha) of semi-natural habitat (exotic forest and pastures), agricultural land and urban land cover was computed in QGIS 3.4.3, based on land-cover data for Terceira Island obtained from [37]. Coordinates of each orchard and landscape composition can be found in Table S1.

### 2.2. Pollinator Sampling

Pollinator sampling was conducted between 17 May 2019 and 5 June 2019, during apple blossom peak, which usually occurs later in Azores than in mainland Portugal. Direct observations were made from 10:00 to 18:00 on days with sunny weather and minimum wind speed. Seven trees per orchard were selected, separated at least by 5 m. Two similar branches per tree were marked for visitation surveys, which consisted of 10 min observation sessions per branch. Visitation rate of flower-visiting insects was estimated once per orchard, recording all visitors, which made contact with the flower stamens. Visitors were identified mostly in situ, and, when not possible to identify them in the field, insects were collected with a pooter for later identification in the lab. In addition to direct observation of flower-visiting insects, pan traps were placed in each orchard in order to account for insect richness at the orchard level. Pan trap surveys were carried out three times during the apple blossoming period per management type. Four yellow pan traps were evenly distributed throughout each orchard and placed directly on the ground, then collected after 7 h at the end of each sampling session. Pan traps were not left for 24h or more as in other studies [20] in order to avoid loss of specimens due to bird ingestion. Moreover, some studies in the UK have shown that leaving pan traps during 6–7 h performed as well as 24 h with regard to number of insects caught, providing data of sufficient quality for quantitative analysis [38]. Contents were emptied into tubes containingethanol (70%) for preservation. The specimens collected, as well as insects trapped with the pooter, were preserved in the fridge (4 °C) until taxonomical identification in the lab.

Specimens collected were sorted first into morphospecies and later identified to species-level under the supervision of expert taxonomists (Dr. Mário Boieiro and PAVB), following the taxonomic nomenclature in [39]. When species-level identification could not be resolved, individuals were identified to the lowest taxonomic unit possible (genus or family). Voucher specimens and a reference collection were deposited in EDTP—Entomoteca Dalberto Teixeira Pombo, University of Azores, Angra do Heroismo, Portugal. All species were classified as indigenous or exotic species. Indigenous species may be endemic (i.e., found only in the Azores) or native nonendemic (i.e., species that colonized the Azores by natural long-distance dispersal mechanisms). Exotic species are those whose original distribution range did not include the Azores and are believed to have arrived as a consequence of human activities; these species often have a cosmopolitan distribution [39].

### 2.3. Pollination Service Provision

Fruit production in orchards was measured as the proportion of flowers on branches that produced fruit (fruit set). The number of flowers was counted on the selected two branches per tree used for visitation observations. The number of fruits on branches was then recorded in early September, before fruit harvest. No chemical thinning was done prior to fruit counts in any orchard, and any hand thinning avoided our marked branches. Some authors have also quantified “initial” fruit set (fruit set two or three weeks after petal fall). Initial fruit set is considered the best indicator of pollination success, as it is measured before fruits are lost to pests. Nevertheless, fruit set at harvest (September onwards) is more relevant for orchard managers [40]. We also measured seed set (number of seeds per apple) on 30 apples per orchard of the most common apple variety, Rennet. Other measures of fruit quality such as weight were also measured on the collected apples (*n* = 30).

### 2.4. Data Analysis

Linear mixed models (LMMs) were used to test for differences in total pollinator visitation frequency (number of visits/open flower in 10 min) and visitation frequency of specific pollinator groups among orchards. We set management type as a fixed predictor of visitation frequency and orchard ID as a random effect. In addition to management, we included three additional landscape variables in the analyses, including area of forest, pastures and agriculture. Because of strong multicollinearity among these variables, we performed a PCA to reduce the land cover variables into a number of orthogonal/uncorrelated axes. We then used the two first principal components (PC1 and PC2) as predictors in the models. PC1 was negatively associated with pastures and positively associated with agricultural areas and to a lesser extent with forest. PC2 was negatively associated with forest and positively with agriculture and pastures. A full description of the PCA results is reported in Supplementary Materials (Figure S1 and Table S2). The possible difference in number of flowers per branch between conventional with herbicides, conventional without herbicides and organic orchards was tested with a Poisson generalized linear mixed model (GLMM) using management type as a predictor and orchard ID as a random effect.

Species diversity was quantified using Hill numbers, also called “the effective number of species”, i.e., the number of equally abundant species that are needed to give the same value of a diversity measure. Hill numbers represent the best choice to quantify abundance-based species diversity [41], and are increasingly used to characterize the taxonomic, phylogenetic or functional diversity of an assemblage. The family of Hill numbers diversity indexes differ among themselves only by the parameter *q* (where *q* controls the weights of species relative abundances), and encompass the three most useful diversity measures: species richness (diversity of order *q* = 0), Shannon diversity (*q* = 1, i.e., the exponent of Shannon entropy), and Simpson diversity *(q* = 2, i.e., the inverse of the Simpson concentration index) [41]. We also calculated rarefied species richness, which was computed to the minimum number of individuals in a sample using the “vegan” package in R. Individual-based rarefaction allows for meaningful standardization and comparison of management systems [42]. Only the main four insect pollinator orders (Coleoptera, Diptera, Hymenoptera and Lepidoptera) were included in the analyses. We tested for the effect of agricultural management on orchard insect abundance, rarefied richness and exponential Shannon–Weaver index (second Hill number) through a negative binomial GLMM and LMMs, respectively (data collected through pan traps). We set management type and landscape composition (PC1 and PC2, see above) as fixed effects and orchard ID as a random effect. Complementarily, we calculated additional consecutive Hill numbers and plotted them as a continuous function of the parameter *q* (ranging from 0 to 5), according to management. This diversity profile characterizes the species abundance distribution of an assemblage and provides complete information about its diversity. The steepness of its slope graphically illustrates the degree of dominance in the assemblage [43].

To test for differences in fruit set, number of seeds per apple (the metrics used for pollination success), and apple quality (weight) we used GLMM models with a binomial (for fruit set) and negative binomial distribution (for seed set and weight).

All GLMMs were graphically validated to ascertain goodness of fit [44] and were followed by a post-hoc test to establish the statistical significance of the variables of interest. All statistical analyses were performed with R statistical software version 5.3.1, using the packages “lme4”, “nlme”, “vegan”, “hillR” and “car” [45].

## 3. Results

### 3.1. Species Composition

We sampled a total of 38 insect species and morphospecies visiting apple orchards, belonging to four arthropod orders, namely, spiders (Arachnidae: Araneae), and insects (Hexapoda: Coleoptera, Diptera and Hymenoptera) (Table S3). The most common group was Hymenoptera, both in visitation frequencies (78.1% of total visits) and regarding individuals collected in pan traps (67.4%). The second most common group was Diptera, with 21.2% of total visits and 28.6% of individual in pan traps. Minor groups include Coleoptera with 0.7% of total visits and 1.9% of individual collected, and the Araneae with no visitations registered but 2.1% of total individuals collected in pan traps. The most common species in visitation surveys were *Apis mellifera* (44.7% of total visits) and *Bombus terrestris* (24.7%) (both Hymenoptera: Apidae), followed by *Episyrphus balteatus* (Diptera: Syrphidae) (6.2%) and *Stomorhina lunata* (Diptera: Calliphoridae) (5.1%). On the contrary, the most common species collected in pan traps were *Halictus morio* (Hymenoptera: Halictidae) (58.3%) and *Sepsis* sp. (Diptera: Sepsidae) (16.8%), followed by *Halictus minutissimus* (Hymenoptera: Halictidae) (4.9%) and *Calliphora vicina* (Diptera: Calliphoridae) (3.4%). Across management systems, *Apis mellifera* and *Bombus terrestris* were dominant in visitation frequencies. However, the most abundant species from pan traps were contrastingly different among management systems (Table S4).

The majority of recorded visiting insects were introduced (73.4%) and a minority was native or endemic (26.5%). In contrast, a majority of insects in pan traps were native or endemic (89.2%) and only a small percentage was introduced (6.6%). However, these proportions showed significant statistical differences across management types, both for visiting insects (χ^2^ = 63.1, df = 2, *p* < 0.0001) and pan traps (χ^2^ = 172.4, df = 2, *p* < 0.0001). Conventional orchards without herbicides showed similar proportions of native and introduced visiting insects, in contrast with the dominance of introduced species in both conventional and organic orchards (Table 1). Lastly, the number (species richness) of native and introduced species also varied according to management: a higher richness of native visiting species was detected in conventional without herbicides and organic orchards in comparison to conventional with herbicides sites. In pan traps, the richness of native species was found to be higher in conventional without herbicides, but similar to conventional with herbicides, while organic orchards presented a lower richness of native species (Table 1).

### 3.2. Effect of Management on Insect Visitation Rates

**Figure 2:**
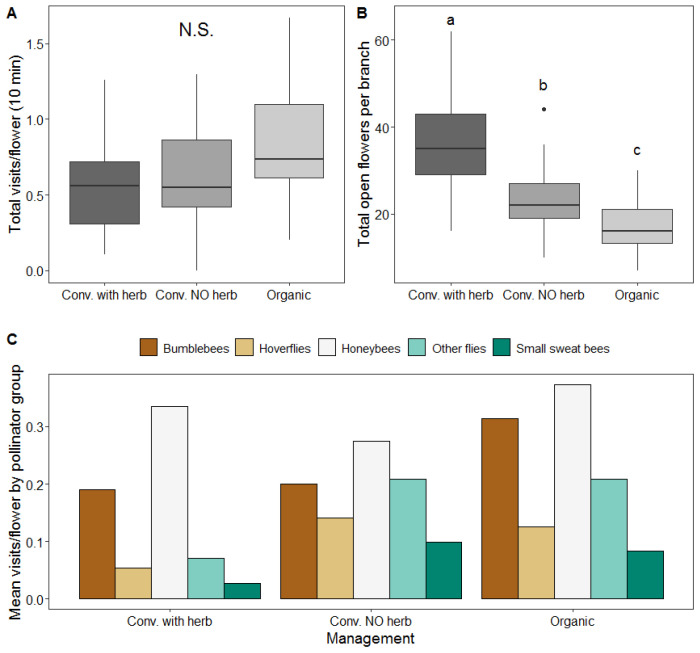
(**A**) Total insect visits per open flowers in 10 min observations according to management; (**B**) Number of open flowers per branch according to different management systems; (**C**) Mean visits per flower for main pollinator groups according to management. N.S. denotes non-significant effects, and different letters represent significant effects between levels. The black horizontal line is the median, the box is defined by the 25th and 75th percentiles (lower and upper quartile) and the whiskers are 1.5 the interquartile range. Data beyond the end of the whiskers plotted individually are outlier points.

Slightly higher values of total visits per open flower were recorded in organic orchards (Figure 2A). However, no statistical differences were found between management systems (χ^2^ = 4.406, df = 2, *p* = 0.110, Figure 2A). In contrast, the effect of landscape composition represented by PC1 had a statistically significant effect on visitation rate (χ^2^ = 4.736, df = 1, *p* = 0.03). The direction of the effect of PC1 was negative (estimate −0.755), indicating a negative association of visitation rates with high amounts of agricultural land cover around orchards. When testing for a possible interaction effect of the taxonomic order (Coleoptera, Diptera or Hymenoptera) or the main pollinator group (honeybees, bumblebees, hoverflies, other flies or small sweat bees) with management system on visitation frequency, we found no significant statistical differences (χ^2^ = 4.42, df = 2, *p* = 0.11 and χ^2^ = 10.62, df = 10, *p* = 0.388 respectively). Mean visits per flower of main pollinator groups are shown in Figure 2C.

Flower count of the assessed branches revealed significant differences in the number of open flowers per branch between conventional with herbicides (36.6 ± 11.2; mean ± SD) and either organic orchards (18.2 ± 6.2), or conventional orchards without herbicides (23.5 ± 7.6) (χ^2^= 84.03, df = 2, *p* ≤ 0.0001, Figure 2B).

### 3.3. Effect of Management on Insect Abundance and Diversity

**Figure 3:**
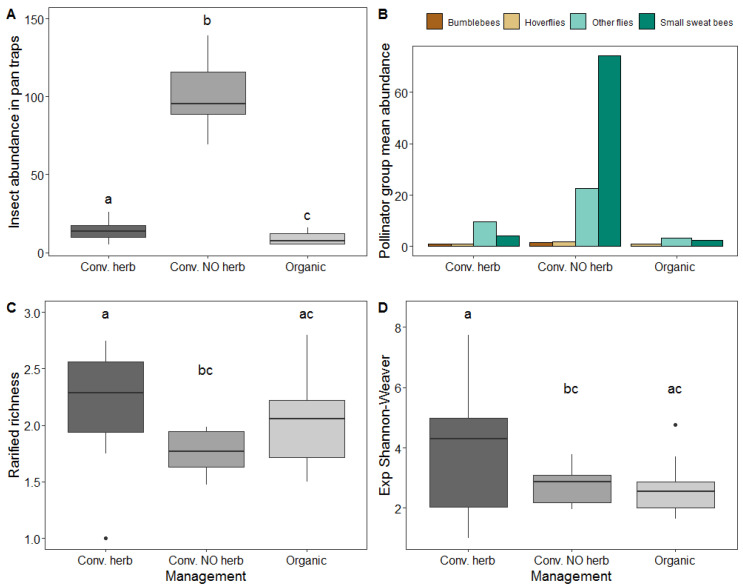
(**A**) Total insect abundance in pan traps in different management systems, (**B**) Mean abundance of main pollinator groups across management, (**C**) Rarified species richness (at the pan trap level) and (**D**) Exponential Shannon–Weaver diversity index or second Hill number (pan trap level) according to management system. N.S. denotes non-significant effects, and different letters represent significant effects between levels. The black horizontal line is the median, the box is defined by the 25th and 75th percentiles (lower and upper quartile) and the whiskers are 1.5 the interquartile range. Data beyond the end of the whiskers plotted individually are outlier points.

Results from the data collected through pan traps showed a statistically significant effect of management on insect abundance (χ^2^ = 102.3, df = 2, *p* ≤ 0.0001, Figure 3A). Significantly higher abundance was found in conventional orchards without herbicides with respect to both organic and conventional orchards with herbicides (Figure 3A). Interaction of management with main pollinator group was significant (χ^2^ = 42.3, df = 5, *p* ≤ 0.0001, Figure 3B), while the effect of PC1 and PC2 were not statistically significant (χ^2^ = 0.79, df = 1, *p* = 0.37 and χ^2^ = 0.46, df = 1, *p* = 0.5).

Management showed a statistically significant effect on rarified richness (χ^2^ = 6.38, df= 2, *p* = 0.04, Figure 3C), and the effect of PC1 was almost significant (χ^2^ = 3.12, df = 1, *p* = 0.08). Rarified richness was found to be higher in the conventional orchards with herbicides with respect to conventional without herbicides, whereas there were no differences with respect to organic orchards (Figure 3C). We also found a statistically significant effect of management on the second Hill number (exponential Shannon–Weaver) (χ^2^ = 7.466, df = 2, *p* = 0.02), being again higher in conventional orchards with herbicides with respect to conventional without herbicides (Figure 3D). The effect of PC1 on the second Hill number was significant (χ^2^ = 4.055, df = 1, *p* = 0.04) and had a positive effect.

**Figure 4:**
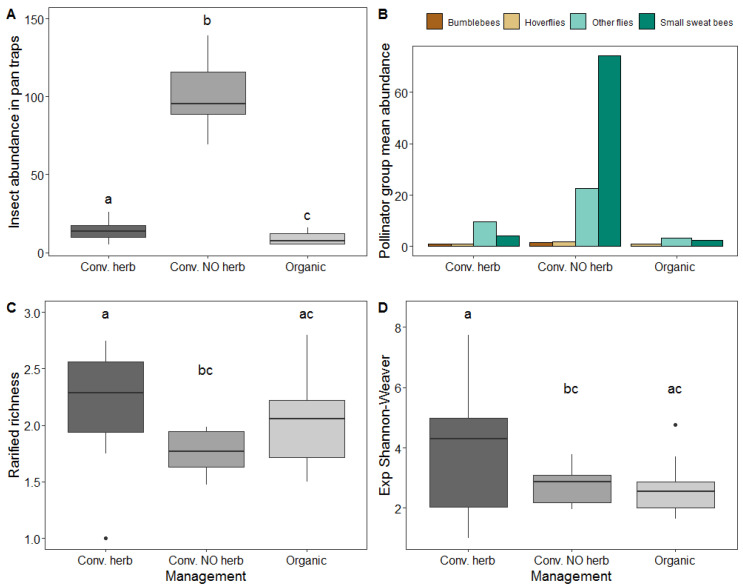
Diversity (Hill numbers) profile for the different management orchards. The *x*-axis is the order *q*, 0 ≤ *q* ≤ 5. The *y*-axis is the Hill number (the effective number of equally abundant species).

The results from comparing the diversity (Hill numbers) profiles between management types showed contrasting patterns in evenness. Organic and conventional orchards with herbicides present a moderately uneven species diversity profile, while conventional without herbicides showed a highly uneven dominance profile, with a very steep decline in the profile curve (Figure 4).

### 3.4. Effect of Management on Pollination Services

**Figure 5:**
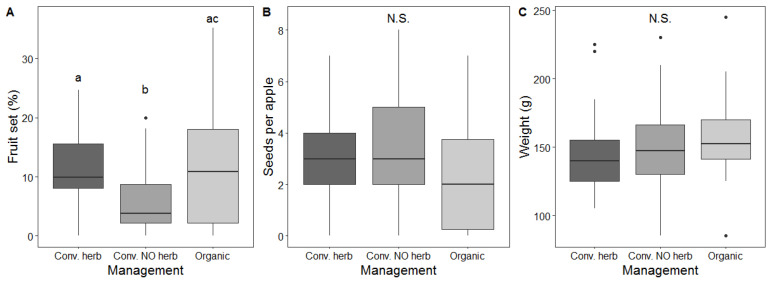
(**A**) Fruit set (number of fruits per flower in each branch) according to management system; (**B**) Seed set (number of seeds per fruit) in different orchards (*n* = 30 per orchard); (**C**) Weight of apples (*n* = 30) according to management system. N.S. denotes non-significant effects, and different letters represent significant effects between levels. The black horizontal line is the median, the box is defined by the 25th and 75th percentiles (lower and upper quartile) and the whiskers are 1.5 the interquartile range. Data beyond the end of the whiskers plotted individually are outlier points.

Management had a statistically significant effect on fruit set (χ^2^ = 7.75, df = 2, *p* = 0.02, Figure 5A), together with landscape variable PC1 (χ^2^ = 4.35, df = 1, *p* = 0.04), which showed a positive estimate effect. On the contrary, the effect of management was not statistically significant on either seed set (χ^2^ = 2.72, df = 2, *p* = 0.257, Figure 5B) or fruit weight (χ^2^ = 0.13, df = 2, *p* = 0.937, Figure 5C). No significant effect of landscape variables was found for either seed set or apple weight.

## 4. Discussion

A caveat of our study was the low sample size, which nonetheless provides insights into island ecology and valuable information for future meta-analysis. Studies such as this one is important within the context of oceanic islands, where information on pollination drivers in agroecosystems is still very scarce. We advocate that data on pollination services at local scales such as those collected in this study are much needed for implementing effective conservation strategies. Given the high costs of fieldwork, future studies could combine appropriate fieldwork and predictive modelling methodologies [46]. This would enhance the generality of results collected at selected sites and make them applicable at larger spatial scales. Furthermore, setting up new field studies, similar to the one carried out here, within long-term monitoring schemes as proposed in [47], would help to quantify temporal changes in pollination services on islands. We discuss the main results of this work in the light of these considerations.

### 4.1. Species Composition in Apple Orchards

Our results indicated that hymenopteran insects dominate apple flower visitation, particularly honeybees and bumblebees, although dipteran species, particularly hoverflies, provided a sizeable portion of our visitation samples. This result is in line with the increasing evidence that other hymenopteran bee species and hoverflies, in addition to widespread honeybees, represent valuable complementary pollination vectors for apple [48]. It has been shown that wild bees are important for global crop pollination including for apples [49,50], and that non-bee pollinators, such as hoverflies, can play an important role in some crops as both pollinators and pest control agents [51].

Our results also indicated that introduced species dominate the insect community visiting apple flowers and that, although native or endemic species are abundant in apple orchards, they might have a lower contribution to apple pollination. These results are in accordance with previous studies showing that native pollinators are abundant and spread across the landscape in Terceira Island [32]. However, we observed significant differences in the relative abundance and species richness of native and introduced species in relation to the management implemented. In organic and conventional orchards with herbicides there was a clear dominance of introduced species visiting apple flowers, whereas in conventional without herbicides the relative abundances were almost equivalent (Table 1). Moreover, the richness of native species visiting apple flowers and sampled in pan traps was higher in conventional orchards without herbicides as compared to the other management types. This could be a result of the large quantity of beneficial flowering plant species present in the herbaceous cover, particularly white clover, *Trifolium repens*, which is highly valuable as pollen source for long-tongued pollinator species [52]. Underground cover was absent in conventional orchards that applied herbicides as expected. In the selected organic orchards, the herbaceous cover was evidently present, but mostly dominated by *Plantago* species, which are mainly wind-pollinated and thus not expected to attract pollinator insects [53]. This result suggests that the type of herbaceous vegetation cover is of paramount importance for the maintenance of a diverse native pollinator load.

### 4.2. Effect of Management on Apple Pollination

Results from this study indicated that management did not have a significant impact on insect visitation rate to apple flowers. These findings are in line with those from other studies in apple [19] and other perennial crops [18], which found no effect of management on pollinator visitation. However, evidence from a large-scale study found contrasting results, with higher flower visitation rates in organic apple orchards vs. integrated pest management sites. Inconsistencies among studies may be explained by the fact that perennial crops usually possess a range of permanent structural elements such as hedgerows that can potentially attract pollinators regardless of the management implemented, thus being ultimately less impacted in terms of floral resources than annual crops [19]. Apple orchards in Terceira Island were all surrounded by diverse structural elements such as hedges and stonewalls that could serve as nesting habitats and attract pollinators even in orchards that applied herbicides. Additionally, the effect of landscape composition around orchards could modulate that of management. We found that visitation rates were negatively associated with higher amounts of agricultural land around orchards. This result is in agreement with previous studies, which have showed that a predominance of agricultural habitats surrounding apple orchards decreases wild bee richness, abundance and diversity [28,54,55,56,57,58]. Therefore, the effects of management on pollination visitation rates seem to depend to a large extent on habitat composition at the landscape level, and could be further modulated by the existence of within-field habitat heterogeneity at the local scale [48].

By contrast, our results suggested that management, together with landscape composition, could have a significant impact on insect abundance, species richness and diversity at the orchard scale. Some studies had previously reported an effect of management on pollinator species richness and diversity in apple orchards [26,28], whereas others had found a non-significant effect [20,27]. In this work, conventional orchards without herbicides had a higher species abundance than conventional with herbicides and, unexpectedly, than organic orchards. This latter result could be explained by the more beneficial underground herbaceous cover with respect to organic orchards as discussed in the previous section. Other studies in cider-apple orchards have shown that plant species richness and the abundance of some plant species in the groundcover favored the presence of pollinators assemblages [53]. Despite their greater insect abundance, conventional orchards without herbicides showed a lower species richness (rarefied) and diversity than conventional with herbicides sites, contrary to our initial expectations. These orchards also showed a highly uneven species diversity profile, i.e., they host an insect community in which species differ widely in their abundance. This highly uneven distribution can lead to a lower species richness after rarefaction, in contrast with the other management systems that showed a more even distribution. It has been shown that when communities are highly uneven, or there is extreme dominance by one or a few species, they could be less resistant to environmental stress [59]. In addition, populations of rare species are more likely to experience local extinctions [60].

Last, our results indicated that management may have a weak impact on pollination service provision, showing a significant effect exclusively on fruit set. In fact, the effect of management on pollination services at local scale appears to be erratic [19]. Organic management has been shown to increase pollination in annual systems like strawberries [61], but evidence on its effects in perennial crops like apple is still inconsistent [48]. Here, management seems insufficient for the improvement of overall pollination services in apple, although further studies undertaking hand pollination and bagging experiments would be needed in order to draw more general conclusions.

## 5. Conclusions

This study showed support for the increasing evidence that hymenopteran bee species and hoverflies, in addition to widespread honeybees, represent important pollination vectors for apple, further calling attention to the need of preserving wild pollinators in agroecosystems. In addition, results from this work suggest that management has a limited effect on apple pollination on an oceanic island, showing no significant effect on pollinator visitation rates and ultimately overall pollination services. On the contrary, a negative effect of increasing surrounding agricultural area on pollinator visitation was observed. Although conventional orchards without herbicides seem to benefit insect abundance at the orchard scale, together with favouring native species, they also showed a very uneven species distribution, leading to lower species richness and diversity than conventional orchards with herbicide application. These findings indicate that the effect of management in apple pollination may depend largely on habitat composition at the landscape level. Nevertheless, further studies are needed to gain a better understanding of the effect of these drivers on insect pollination in apple and other agroecosystems.

## Figures and Tables

**Table 1:** Total number of visits or number of individuals in pan traps (*n*), and number of species (S) of native/endemic and introduced species in different management orchards.

Management	Insect Visitation				Pan Traps			
	Native/Endemic		Introduced		Native/Endemic		Introduced	
	N	S	N	S	N	S	N	S
Conventional with herbicides	93	6	457	6	84	11	24	5
Conventional NO herbicides	162	11	216	5	737	15	36	5
Organic	87	11	274	4	62	8	5	4

## Supplementary Materials

The following are available online at https://www.mdpi.com/2075-4450/11/6/351/s1, Table S1: Geographical coordinates for the six study orchards and landscape composition, Table S2: Results from the principal component analysis, Table S3: List showing total number of visits to apple flowers (timed counts) and number of individuals found in pan traps per insect species/morphospecies in each individual orchard, Table S4: List showing total number of visits to apple flowers (timed counts) and number of individuals found in pan traps per insect species/morphospecies across management systems, Figure S1. Graph showing results from the principal component analysis (PCA) performed on the three land cover variables.
