# Supplementary material for: Limited Effect of Management on Apple Pollination: A Case Study from an Oceanic Island
Source: Insects. 2020 Jun 4;11(6):351. doi: 10.3390/insects11060351 (PMC7349632; doi:10.3390/insects11060351)
Supplement: Supplementary file 1 [file insects-11-00351-s001.pdf]

Supplementary materials

Table S1. Geographical coordinates for all orchards in Terceira Island, with corresponding management type, and landscape composition (amount of agricultural, pasture and exotic forest, in hectares).

**Orchard**	**ID**	**Management**	**Latitude**	**Longitude**	**Agricultural**	**Pasture**	**Exotic forest**
1	TER-BISC-T-601	Conventional with herbicides	38.77344909	-27.25435592	24.72	24.8	50.48
2	TER-BISC-T-602	Conventional with herbicides	38.77550157	-27.26538990	8.750	34.0	57.24
3	TER-BISC-T-603	Conventional NO herbicides	38.77874295	-27.27064368	38.44	20.91	40.65
4	TER-BISC-T-606	Conventional NO herbicides	38.78102385	-27.25824487	42.31	19.9	37.78
5	TER-QUAT-T-604	Organic	38.78413456	-27.22568350	6.05	65.52	26.7
6	TER-QUAT-T-605	Organic	38.78796208	-27.22176542	15.06	66.89	16.75

Table S2. Results from the principal component analysis (PCA), showing proportion of variance explained by the axis scores (PC1 and PC2) and correlation with the original land cover variables.

	Proportion of variance	Cumulative proportion	Exotic forest	Agriculture	Pasture
PC1	0.7294	0.7294	0.001086741	0.706562784	0.707649526
PC2	0.2706	1	-0.8164959	0.4091891	0.4073068

Table S3. Total number of visits to apple flowers (N° of visits, timed counts) and number of individuals found in pan traps (N pan traps) per insect species/morphospecies in each orchard.

Order	Family	Species	Colonization Status	Conventional with herbicides				Conventional NO herbicides				Organic			
				Orchard 1		Orchard 2		Orchard 3		Orchard 4		Orchard 5		Orchard 6	
				N° of visits	N pan traps	N° of visits	N pan traps	N° of visits	N pan traps	N° of visits	N pan traps	N° of visits	N pan traps	N° of visits	N pan traps
Araneae	Lycosidae	*Pardosa acorensis*	Endemic	0	0	0	0	0	0	0	0	0	0	0	21
Coleoptera	Apionidae	*Aspidapion radiolus*	Introduced?	0	0	0	0	0	3	0	0	0	0	0	0
	Chrysomelidae	*Phyllotreta* sp.	Introduced?	0	1	0	0	0	0	0	0	0	0	0	0
	Curculionidae	*Gymnetron pascuorum*	Introduced	0	0	0	0	0	0	0	0	0	1	0	1
	Nitidulidae	*Meligethes aeneus*	Introduced	0	0	1	0	5	0	0	0	0	1	0	0
	Phalacridae	*Stilbus testaceus*	Native	0	0	0	2	0	0	3	0	0	0	0	0
	Scraptiidae	*Anaspis proteus*	Macaronesia	0	2	0	4	0	1	0	2	0	0	0	0
	Staphylinidae	*Rugilus orbiculatus*	Native	0	0	0	0	0	1	0	0	0	0	0	0
Diptera	Anthomyiidae	MF64	-	0	1	0	0	0	0	0	0	1	0	0	0
		MF68	-	0	0	0	0	0	0	0	0	0	0	0	0
		MF81	-	0	0	0	0	0	0	0	0	0	0	0	3
	Calliphoridae	*Calliphora vicina*	Introduced	1	8	3	9	0	10	8	7	6	0	0	0
		Pollenia rudis	Introduced	3	3	0	0	1	5	0	7	5	1	17	0
		Stomorhina lunata	Native	13	0	0	0	10	0	25	1	11	0	4	0
	Chamaemyiidae	*Chamaemya* sp.	Native	0	1	0	0	0	0	0	0	0	0	0	0
	Dolichopodidae/Agromyzidae	MF67	-	0	0	0	1	0	1	0	3	0	0	0	0
	Muscidae	*Phaonia subventa*	Introduced	0	0	1	0	0	0	0	0	0	0	0	0
		Stomoxys calcitrans	Introduced	0	0	0	0	0	0	0	2	0	1	0	0
	Sarcophagidae	MF76	Introduced	0	2	0	0	0	2	0	0	0	0	0	0
	Scathophagidae	*Scathophaga litorea*	Native	0	1	0	0	0	0	1	0	0	0	1	0
	Sepsidae	*Sepsis* sp.	Native	4	17	15	32	13	65	18	42	3	4	10	6
	Syrphidae	*Episyrphus balteatus*	Native	6	1	8	0	25	2	6	0	14	0	21	0
		Eristalis arbustorum	Native	0	0	0	0	1	1	0	4	0	0	1	0
		Eristalis tenax	Native	0	0	0	0	1	0	0	2	0	1	0	0
		Meliscaeva auricollis	Native	0	0	0	0	0	0	0	0	1	0	0	0
		Syritta pipiens	Native	2	0	2	1	0	0	0	0	7	0	2	0
		Xylota segnis	Native	0	0	0	0	0	2	0	2	0	1	0	0
Hymenoptera	Apidae	*Apis mellifera*	Introduced	55	0	259	0	90	0	18	0	67	0	88	0
		Bombus terrestris	Introduced	59	1	75	0	49	3	45	0	54	0	37	0
	Formicidae	*Lasius grandis*	Native	34	2	2	0	14	4	15	7	0	0	0	9
		MF73	-	0	0	0	2	0	0	0	1	0	0	0	0
	Halictidae	*Halictus minutissimus*	Native	0	2	0	3	0	6	0	36	0	0	0	1
		Halictus morio	Native	1	5	7	9	20	276	7	270	3	12	1	5
		Halictus villosulus	Native	0	0	0	2	0	5	1	1	0	2	1	0
	Ichneumonidae	MF79	-	0	1	0	0	0	0	0	0	0	0	0	0
	Megachilidae	*Megachile centuncularis*	Native	0	0	0	0	0	0	0	1	0	0	0	0
	Vespidae	*Ancistrocerus parietum*	Native	0	0	0	0	0	0	0	3	1	0	1	0
		Vespula germanica	Native	0	0	0	0	0	0	2	0	0	0	1	0

Table S4. Total number of visits to apple flowers (timed counts, N° of visits) and number of individuals found in pan traps (N pan traps) per insect species/morphospecies in each orchard management type: conventional with herbicides, conventional without herbicides and organic.

Order	Family	Species	Colonization Status	Conventional with herbicides		Conventional NO herbicides		Organic	
				N° of visits	N pan traps	N° of visits	N pan traps	N° of visits	N pan traps
Araneae	Lycosidae	*Pardosa acorensis*	Endemic	0	0	0	0	0	21
Coleoptera	Apionidae	*Aspidapion radiolus*	Introduced?	0	0	0	3	0	0
	Chrysomelidae	*Phyllotreta sp.*	Introduced?	0	1	0	0	0	0
	Curculionidae	*Gymnetron pascuorum*	Introduced	0	0	0	0	0	2
	Nitidulidae	*Meligethes aeneus*	Introduced	1	0	5	0	0	1
	Phalacridae	*Stilbus testaceus*	Native	0	2	3	0	0	0
	Scraptiidae	*Anaspis proteus*	Macaronesia	0	6	0	3	0	0
	Staphylinidae	*Rugilus orbiculatus*	Native	0	0	0	1	0	0
Diptera	Anthomyiidae	MF64	-	0	1	0	0	1	0
		MF68	-	0	0	0	31	0	0
		MF81	-	0	0	0	0	0	3
	Calliphoridae	*Calliphora vicina*	Introduced	4	17	8	17	6	0
		Pollenia rudis	Introduced	3	3	1	12	22	1
		Stomorhina lunata	Native	16	0	35	1	15	0
	Chamaemyiidae	*Chamaemya* sp.	Native	0	1	0	0	0	0
	Dolichopodidae/Agromyzidae	MF67	-	0	1	0	4	0	0
	Muscidae	*Phaonia subventa*	Introduced	1	0	0	0	0	0
		Stomoxys calcitrans	Introduced	0	0	0	2	0	1
	Sarcophagidae	MF76	Introduced	0	2	0	2	0	0
	Scathophagidae	*Scathophaga litorea*	Native	0	1	1	0	1	0
	Sepsidae	*Sepsis* sp.	Native	19	49	31	107	13	10
	Syrphidae	*Episyrphus balteatus*	Native	14	1	31	2	35	0
		Eristalis arbustorum	Native	0	0	1	5	1	0
		Eristalis tenax	Native	0	0	1	2	0	1
		Meliscaeva auricollis	Native	0	0	0	0	1	0
		Syritta pipiens	Native	4	1	0	0	9	0
		Xylota segnis	Native	0	0	0	4	0	1
Hymenoptera	Apidae	*Apis mellifera*	Introduced	314	0	108	0	155	0
		Bombus terrestris	Introduced	134	1	94	3	91	0
	Formicidae	*Lasius grandis*	Native	36	2	29	11	0	9
		MF73	-	0	0	0	1	0	0
	Halictidae	*Halictus minutissimus*	Native	0	5	0	42	0	1
		Halictus morio	Native	4	14	27	546	8	17
		Halictus villosulus	Native	0	2	1	6	1	2
	Ichneumonidae	MF79	-	0	1	0	0	0	0
	Megachilidae	*Megachile centuncularis*	Native	0	0	0	1	0	0
	Vespidae	*Ancistrocerus parietum*	Native	0	0	0	3	2	0
		Vespula germanica	Native	0	0	2	0	1	0

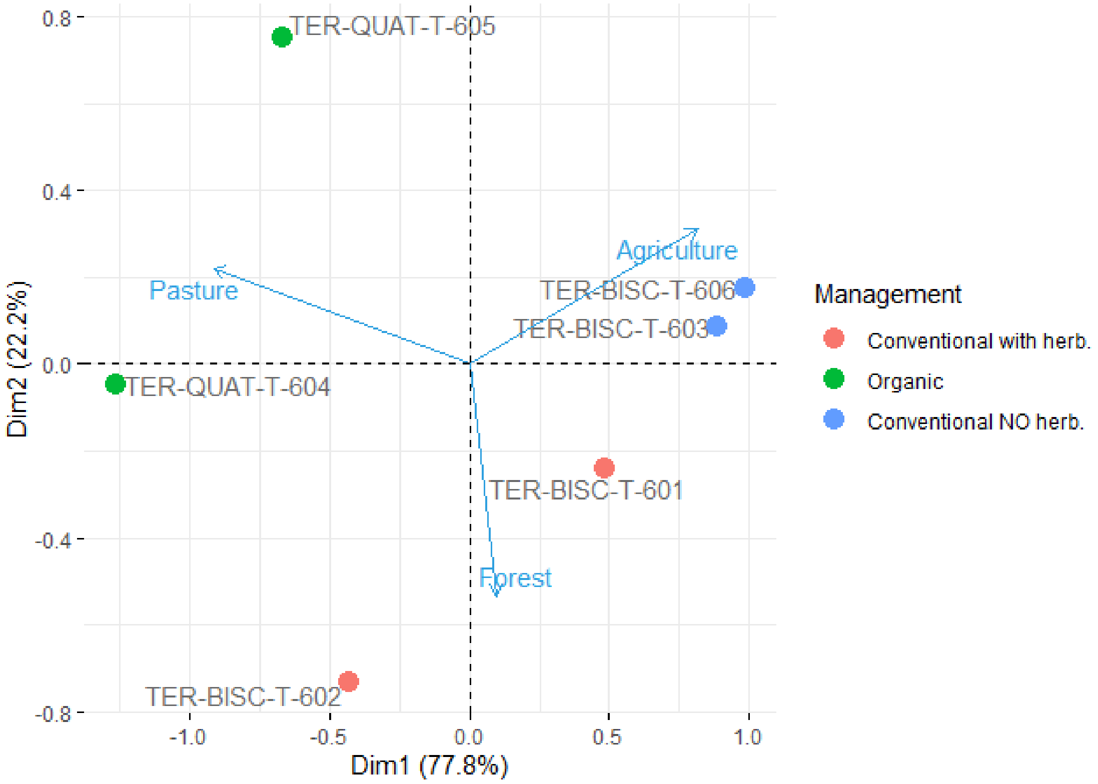

Fig. S1. Graph showing results from the principal component analysis (PCA) performed on the three land cover variables. Site scores are coloured according to management system.
